# Self-efficacy of clinical performance in nursing students and its relationship with the motivation of field choice and clinical education status

**DOI:** 10.1186/s12909-025-07372-8

**Published:** 2025-05-24

**Authors:** Maryam Momeni, Mohammadreza Asadi, Haniyeh Shadin, Sajad Noorian, Mojtaba Senmar

**Affiliations:** 1 Social Determinants of Health Research Center, Research Institute for Prevention of Non-Communicable Diseases, Qazvin University of Medical Sciences, Qazvin, Iran; 2 Student Research Committee, Qazvin University of Medical Sciences, Qazvin, Iran; 3 Department of Statistics, Faculty of Science, University of Qom, Qom, Iran

**Keywords:** Self-efficacy, Motivation, Nursing student, Education

## Abstract

**Introduction:**

Clinical performance self-efficacy is an important element in providing safe care, and it has an important place in nursing education. the self-efficacy of clinical performance can be affected by various factors such as motivation and clinical education status. Therefore, the present study was conducted to investigate the self-efficacy of clinical performance and its relationship with the motivation of choosing a field of study and the status of clinical education in nursing students.

**Methods:**

This descriptive cross-sectional study was conducted in 2024 among 237 nursing students at Qazvin University of Medical Sciences, located in the northwest of Iran. The sample population of this study included in the study by census method. The data collection tools in this study included a demographic information checklist, clinical performance self-efficacy scale, study field motivation Questionnaire, and clinical education status Questionnaire.

**Results:**

The results showed that the self-efficacy of clinical performance with mean of 127.88 ± 21.79 is at the desired level. Motivation was at a good level with mean of 40.50 ± 8.44. The results also showed that the state of clinical education with mean of 100.27 ± 21.90 is at the average level. The results of the multivariate regression analysis showed that, with an increase of one unit of the motivation to choose a field, the self-efficacy of clinical performance increases by mean of 0.985 units (*P* < 0.001), but the state of clinical education does not significantly predict self-efficacy of clinical performance (*P* = 0.294).

**Conclusion:**

Based on the results of this study, self-efficacy of clinical performance and motivation in nursing students is in a favorable and good condition, but the condition of clinical education is at an average level. The results of this study also indicated that motivation for choosing a field of study plays a significant role in predicting self-efficacy of clinical performance. Adopting motivational enhancement approaches and improving the quality of clinical education can contribute to the promotion of self-efficacy in the clinical performance of nursing students.

## Introduction

Nursing is a branch of medical sciences whose mission is to serve humans and provide comprehensive care [1]. Nurses, due to their unique position, apply the majority of their time with patients [2]. In this clinical field, the preparation of nursing students, as future nurses, is vital to provide safe and quality care [3, 4]. They should strive to enhance their scientific capabilities [5]. One of the most important components of nursing education is clinical education [6]. Self-efficacy theory is one of the widely used theories in measuring the degree of confidence in performing clinical skills of students [6].

Self-efficacy is an important element in increasing the self-confidence of nursing students, which will lead to the acquisition of clinical knowledge and skills [7]. Clinical self-efficacy is a judgment about the ability to organize and perform nursing care independently and based on the nursing process at the bedside [8]. The meaning of clinical self-efficacy in students is their beliefs about their abilities in performing tasks assigned to them [9]. This concept is a summary of the goals of nursing education, which include the ability to establish professional communication, having professional knowledge, and the ability to solve complex problems [10].

In the words, motivation means dynamic and movement, and from the organizational point of view, it is an internal factor that causes a change in behavior and movement toward career goals [11, 12]. Motivation is one of the key factors in the learning process, which plays a very important role in nursing education [13]. Choosing a nursing field is influenced by a combination of different motivations such as helping others, a positive nursing image, opportunity, and job security [11]. Although studies show that interest in the field of study improves students'aptitude and clinical qualifications [14]; we are also faced with the fact that the student's lack of interest in the field of study leads to a lack of energy, attention, and enthusiasm, and even to stay away from the classrooms [15]. Therefore, it should be said that motivation has a structure that affects student retention, academic progress, and sustainable behavior [11]. In addition to motivation, Abdal et al.'s study showed that the quality of clinical education is also one of the factors affecting students'clinical self-efficacy [16].

Clinical education is the heart of nursing education, which accounts for almost half of nursing education [7, 17]. Undoubtedly, clinical education is considered the most significant and fundamental component of nursing education programs, as it fosters the professional and academic development of nursing students [18]. In addition to exposing students to the clinical learning environment, clinical education provides an opportunity for them to acquire the ability to translate theoretical knowledge into a range of complex cognitive, affective, and psychomotor skills [19]. It also enables them to deliver optimal health services and professional care to patients [20]. Although clinical learning is influenced by various factors, effective clinical education is considered a prerequisite for providing correct care measures and increasing the quality of clinical skills in students [21].

The state of clinical education in various parts of the world is influenced by a range of variables, including economic, cultural, and healthcare conditions [22]. For example, in South Korea and Iran, inadequate equipment and teaching hospitals, a shortage of qualified clinical instructors, and insufficient evaluations are among the main problems of clinical education [23, 24]. In developed countries such as Australia, the use of advanced equipment and well-equipped educational environments are strengths of this system, but limited support for students in the clinical setting, the number of students in each department, and financial irregularities are important obstacles to effective clinical education [25]. Although, studies show that the quality of clinical education is different for the students of different semesters [26, 27]; However, the clinical environment, individual factors, and the ability of the trainer are among the factors influencing self-efficacy [28]. Therefore, it should be said that the clinical self-efficacy of students is influenced by their clinical education [16].

Studies conducted in Iran indicate that nursing students possess moderate levels of clinical self-efficacy, and this variable is influenced by various individual and environmental factors [16, 29]. Self-efficacy is also affected by various factors such as creativity and a sense of belonging [8, 30]. A study in Turkey revealed that self-efficacy is influenced by both intrinsic and extrinsic motivations [31]. Although a high level of this component leads to confidence in one's abilities and more effort and perseverance [32], and neglecting the improvement of clinical self-efficacy causes a decrease in the quality of trained personnel in the nursing profession [33].

However, we see that there is less focus on the self-efficacy of nursing students'clinical performance [34]. This is even though learning and changing desired behavior in this group is not only influenced by strong and sufficient motivation [35], but also the quality of clinical education can be considered as one of the effective factors in acquiring and improving this self-efficacy. In general, the future of nursing will be in the hands of the students in this field [3]. Students who will be responsible for providing care to people shortly. Fixing the existing deficiencies and improving the self-efficacy of the clinical performance of these students depends on the identification of factors affecting this concept. Considering the insufficiency of information in this field, and the differences in learning environments, educational systems, and facilities available in different parts of the world, the present study was conducted to investigate the self-efficacy of clinical performance and its relationship with the motivation of choosing a field and the status of clinical education in nursing students.

## Materials and methods

### Study design

This descriptive cross-sectional study was conducted among 237 nursing students at Qazvin University of Medical Sciences, located in the northwest of Iran (June to October 2024).

### Participants

Due to the limited sample population, all students were included in the study based on the entry criteria (response rate: 86.29%). Therefore, 233 students participated in the study by census method. The inclusion criteria were enrollment in the 4 th to 8 th semester of the Bachelor of Science in Nursing program and willingness to participate. Questionnaires that were incompletely completed and students who stated that they had a history of mental illnesses were excluded from the study. The questionnaires were completed in the presence of the researcher.

### Data collection tools

#### Part 1: Demographic checklist

A researcher-made checklist was used to collect demographic information. This checklist included information about age, sex, marital status, place of residence, semester, grade point average (GPA), and working experience in the hospital.

#### Part 2: Clinical performance self-efficacy scale

This scale was designed and psychometrically tested by Cheraghi et al. [36]. This scale contains 37 items regarding self-efficacy evaluation of students'clinical performance in 4 areas patient assessment, nursing diagnosis and planning, implementation of care plan, and evaluation of care plan. Answers to items are on a 5-point Likert scale. The maximum score of the scale is 185 and the lowest score is 37. Higher scores indicate greater clinical performance self-efficacy. The validity of this scale in Iranian society has been confirmed by the professors of Hamedan University of Medical Sciences in the form of content validity, and the reliability of this tool has also been confirmed by Cheraghi et al. with a Cronbach's alpha of 0.72 [36]. The reliability of the instrument was confirmed in this study (Cronbach's alpha of 0.73).

#### Part 3: Questionnaire of students'motivation to choose their field of study

This questionnaire was prepared and adjusted by Pouladi and Noroozi [35]. The questionnaire contains 14 items in 3 dimensions financial motivation (7 items), spiritual motivation (3 items), and social motivation (4 items). Answers to items are on a 5-point Likert scale from very high (5) to very low (1). The minimum score of the whole scale is 14 and the maximum score is 70. Thus, the motivation is described in 4 levels: weak (14—< 28), average (28—< 42), good (42—< 56) and very good (56–70). The validity and reliability of this tool was done by Pouladi and Noroozi in Iran and while confirming the validity of this tool, its reliability was confirmed with Cronbach's alpha of 0.7 [35]. The internal reliability of the entire questionnaire in the present study was obtained using Cronbach's alpha of 0.71.

#### Part 4: Questionnaire for examining the clinical education status

The clinical education survey Questionnaire was designed by Mardani Hamuleh et al. [37]. This tool includes 33 items (5 areas) in the form of a 5-point Likert scale ranging from excellent (5 points) to very poor (1 point). The first field, goals and educational program includes 11 items with a score range of 11 to 55. (41 to 55 good levels, 26 to 40 average levels, and 11 to 25 poor levels). The second field of the instructor's performance includes 9 items with a range of scores from 9 to 45 (35 to 45 is a good level, 22 to 34 is an average level, and 9 to 21 is a poor level). The third field of interaction with students includes 4 items with a range of scores from 4 to 20: (16 to 20 at a good level, 10 to 15 at an average level, and 4 to 9 at a poor level). The fourth field of ​​the educational environment includes 5 items with a range of scores. It is 5 to 25 (19 to 25 good level, 12 to 18 medium level, and 5 to 11 poor level). The last field of the monitoring and evaluation includes 4 items with the same score range as the third field. The validity and reliability of the above instrument have been confirmed in a study at Ahvaz University of Medical Sciences. The reliability of the scale was examined by the test–retest method and the correlation coefficient between the two tests was reported to be 0.88 [38, 39]. In the current study, the internal reliability of the questionnaire was good (Cronbach's alpha = 0.87).

### Analysis

The collected data were analyzed by SPSS software version 25 and using descriptive indices such as mean and standard deviation (for quantitative variables), number, and percentage (for qualitative variables). The normality of the data in different groups was checked using the Kolmogorov–Smirnov test. To investigate the relationship between variables, Pearson's correlation coefficient and univariate regression were used; Then, to adjust the effect of confounding variables, multivariate regressions were used. A significance level of 0.05 was considered.

## Results

In total, out of 237 students included in the study, 133 were female (56.1%) and the rest were male. The mean age of the participants in this study was 22.73 ± 2.15. Most (97.0%) of the participants in this study were single people (Table 1).

**Table 1:** Demographic variables of nursing students (*n* = 237)

Variable		N
Gender	Female	133 (56.1)
	Male	104 (43.9)
Marital status	Single	230 (97.0)
	Married	7 (3.0)
Place of residence	Home	132 (55.7)
	Dormitory	105 (44.3)
Semester	4	55 (23.2)
	5	44 (18.6)
	6	44 (18.6)
	7	48 (20.3)
	8	46 (19.4)
Working experience in the hospital	Yes	70 (29.5)
	No	167 (70.5)
GPA (Mean ± SD)		16.69 ± 1.28
Age (Mean ± SD)		22.73 ± 2.15

The results showed that the self-efficacy of clinical performance with mean of 127.88 ± 21.79 is at the desired level. The results of data analysis showed that students'motivation to choose a field of study is at a good level with mean score of 40.52 ± 8.44. The results also showed that the state of clinical education with mean of 100.27 ± 21.90 is at the average level (Table 2).

**Table 2:** Clinical performance self-efficacy, motivation of field choice and clinical education status

Variable		Mean ± SD
Clinical performance self-efficacy	Assessment	41.05 ± 7.56
	Diagnosis and planning	30.26 ± 6.51
	Implementation	36.30 ± 6.24
	Evaluation	20.27 ± 4.24
	Total score	127.88 ± 21.79
Motivation of field choice	Financial	21.51 ± 5.72
	Spiritual	9.18 ± 2.93
	Social	9.81 ± 2.95
	Total score	40.50 ± 8.44
Clinical Education Status	Goals and Educational Program	33.79 ± 8.22
	Instructor's Performance	30.43 ± 7.00
	Interacting with Students	11.78 ± 3.39
	Educational Environment	12.14 ± 4.07
	Monitoring and Evaluation	12.13 ± 4.05
Total score		100.27 ± 21.90

The results of Pearson's test showed that self-efficacy of clinical performance has a moderate positive and significant relationship with the motivation of choosing a field of study (*R* = 0.39, *P* < 0.001) and a weak positive and significant relationship with the state of clinical education (*R* = 0.16, *P* = 0.011). Also, this test showed that there is a weak positive and significant relationship between the state of clinical education and the motivation of choosing a field of study (*R* = 0.26, *P* < 0.001) (Table 3).

**Table 3:** Pearson correlation coefficient test of the relationship between clinical performance self-efficacy, motivation of field choice and clinical education status

Variable		Clinical performance self-efficacy	Motivation of field choice	Clinical Education Status
Clinical performance self-efficacy	Pearson Correlation	1	0.39	0.16
	Sig. (2-tailed)		< 0.001	0.011
Motivation of field choice	Pearson Correlation	0.39	1	0.26
	Sig. (2-tailed)	< 0.001		< 0.001
Clinical Education Status	Pearson Correlation	0.16	0.26	1
	Sig. (2-tailed)	0.01	< 0.001	0.16

For the selection of variables in the regression model, various variable selection methods such as Enter, Forward, Backward, and Stepwise were employed. The consistency of the results was examined, and the best model was selected based on the coefficient of determination (R^2^). It is important to note that only statistically significant variables were included in the final model, while other variables for which the null hypothesis of their coefficients being zero was not rejected were excluded from the final model. Consequently, the regression model does not include variables whose coefficients were found to be statistically insignificant.

The results of univariate linear regression analysis revealed that the self-efficacy of clinical performance (as a dependent variable) is significantly associated with the motivation to choose a field (*P* < 0.001) and the status of clinical education (*P* = 0.011). To adjust the effect of confounding variables, multivariate regression analysis was done. The results of this test showed that with an increase of one unit of the motivation to choose a field, the self-efficacy of clinical performance increases by mean of 0.985 units (*P* < 0.001), but, the status of clinical education does not predict the self-efficacy of clinical performance (*P* = 0.294) (Table 4).

**Table 4:** Regression analysis of the effect of motivation of field choice and clinical education status on clinical performance self-efficacy

Variable’s	Univariate linear regression		Multiple linear regression	
	B	*P*- value	B	*P*- value
Motivation of field choice	1.03	< 0.001	0.98	< 0.001
Clinical Education Status	0.16	0.01	0.06	0.29

## Discussion

This study was conducted to investigate the self-efficacy of clinical performance and its relationship with the motivation of choosing a field and the status of clinical education in nursing students. Based on the findings, self-efficacy of clinical performance and motivation in nursing students is in a favorable and good condition, but the condition of clinical education is at an average level. The findings of this study also indicated that motivation for choosing a field of study plays a significant role in predicting self-efficacy of clinical performance.

### Clinical performance self-efficacy

The findings showed that the self-efficacy of clinical performance of students is at a favorable level; which is in line with the findings of some studies [14, 40]. Kamali et al. [41] showed in a study that the level of clinical self-efficacy of nursing students is higher than average [41]. In interpreting the difference in the results of the two studies, it should be said that Kamali et al. conducted their study only on 5 th-semester nursing students, while the present study has a higher statistical population. Among the fields of the clinical performance self-efficacy scale, the field of implementation of the care program received a higher standard score. This finding is in line with the findings of two studies in Iran [9, 13]. In Khatony et al.'s study (2023) on nursing students [29], the level of this area is lower than the level reported in the present study. In the interpretation of this difference, it should be said that in Khatony et al.'s study, the students of the lower semesters were also included in the study, but in the present study, undergraduate students from the 4 th to 8 th semesters were included in the study.

Based on the results of the present study, the field of patient assessment also received a high standard score, which is in line with the findings of another study in Iran (2023) [32]. Although in Kamali et al.'s study (2023), this area has been given the highest score [41]. However, the mean score is lower than the present study. In the interpretation of this difference, it should be said that the study by Kamali et al. was conducted as an intervention and the study has a lower sample size than the present study. Based on the results of the present study, the evaluation area of ​​the care program obtained a lower score than the two mentioned areas. This is in line with the findings of another study in East Guilan [13]. Contrary to the results of the present study, in the study of Khatony et al. [29], the field of evaluation is in second place [29]. In the interpretation of this difference, it should be noted that there may not be the same criteria for the evaluation of the care program among different educational environments. Although in the present study, the mean score of the nursing diagnosis area is higher than the mean, this area has the lowest score among the self-efficacy areas of clinical performance. Some studies also confirm this finding [29, 41].

### Motivation of field choice

The present study showed that the mean score of motivation in nursing students is in good condition, which is in line with the findings of some studies [13, 42]. This finding is not consistent with the results of a study in China [43]. Also, Mahrous et al. [44] in a study in Saudi Arabia, reported that students'motivation is at an average level [44]. The non-uniformity of the motivation level of nursing students in different studies can be caused by the different educational conditions and environments, and numerous individual and social factors.

Among the areas of the motivation scale, the area of ​​financial motivation received the highest score, which is in line with the findings of Grande et al.'s study (2022) [45]. Contrary to the results of the present study, in the another study in Norway, the scores of internal motivation were higher than other motivations [42]. It should be mentioned that this study was conducted on first-year nursing students. The passage of time and familiarity with the education process can cause internal motivation to decrease. The findings of the present study showed that spiritual motivation also plays an important role in choosing a nursing field, which is in line with the findings of some studies in this field [44, 46]. The present study showed that social motivation has less impact on choosing the nursing field. In contrast to the results of the present study, Kotera et al. [47] in a study in UK showed that this area scored high [47]. In explaining this difference, it can be said that cultural and social structures are among the effective factors in people's motivation.

### Clinical education status

The findings of this study showed that the clinical education status is at an average level. In line with this study, some studies also reported the status of clinical education in an average state [48, 49]. Contrary to the results of the present study, Otaghi et al. (2024), in a study on nursing students during the COVID-19 pandemic, showed that the clinical education status is at a favorable level [50]. Considering that in the study of Otaghi et al. (2024), all students were included in the study, we should take into account the limitation that students studying in lower semesters have a limited view of clinical education and cannot compare this group of students with students who have been in clinical environments for a longer time. Among the domains of the clinical education status scale, the domain of the instructor's performance, despite obtaining the highest score, was placed in an average status. This finding was also reported in another study in Iran [51]. In their study in Turkey, Taylan et al. [52] showed that the performance of the instructor is in a favorable condition from the perspective of nursing students [52]. Establishing effective communication with students and the all-round support of instructors in the clinical environment in the above study has been able to have a positive effect on students'views.

Although in the present study, the field of goals and educational planning also got a high score, it was in an average condition, which is in line with the findings of the study by Emami and et al. (2021) [53]. In another study in Tanzania, nursing students stated that insufficient planning of clinical education hurt their clinical learning [54]. In other words, they did not describe the state of planning of clinical education properly, which is somewhat inconsistent with the results of the present study. It seems that in Jacob et al.'s study, the length of stay in each department and the irregular rotation of shifts were among the reasons that caused this view to be created among students [54]. Among the fields of the clinical education status scale, the field of monitoring and evaluation was in the next rank and was in an average condition. This finding was also reported in the study of Khedmatizare et al. [55], But Seidi et al. (2023) declared the level of this area to be in a favorable condition [51]. In this study, the two fields of interaction with students and the educational environment received the lowest scores compared to other fields. This finding is in line with the findings of some studies in this field [51, 56]. Getie et al. [57] also showed that from the point of view of nursing graduates in Ethiopia, the clinical environment is not suitable for effective clinical education [57]. From the point of view of students; An environment suitable for learning respects students and gives them the right opportunity to learn and achieve their goals [51]. It seems that individual, structural, and social differences can create different results [58].

### Relationships between variables

The present study showed that motivation for choosing a field of study plays a significant role in predicting self-efficacy of clinical performance. No studies related to this topic were found in the researchers'searches. However, consistent with the findings of the present study, two studies conducted in Iran reported that clinical performance self-efficacy and motivation have a positive and significant relationship with each other [13, 15]. Bandura states that self-efficacy beliefs affect students'thinking, behavior, and motivation. This means that students who have a high level of self-efficacy have a more positive attitude toward the results of their work, and as a result, a high level of self-efficacy increases a person's internal motivation [59]. Therefore, it should be said that self-efficacy is a personal construct that is influenced by behaviors and environmental/social variables and influences them [60]. The findings of the current study also confirmed this interpret. Based on the findings of the present study, the status of clinical education does not have a significant role in predicting self-efficacy of clinical performance.

Although no study was found that specifically examined the predictive role of clinical education status on clinical performance self-efficacy, the study conducted by Drisfard et al. [61] showed that there is no significant relationship between the perception of self-efficacy and the state of clinical education [61]. In another study on nursing students (interventional study), Zarshenas et al. [62] reported that the use of a micro-learning teaching method to improve nursing students'clinical skills has a significant effect on their self-efficacy [62]. Although the above study did not consider the status of clinical education. However, it should be noted that modern teaching methods can be part of the quality of clinical education. In explaining this difference, it should be said that the above study was conducted among community health internship students. Meanwhile, the current study has included different academic semesters. Apart from this, the above intervention study considers a part of clinical skills. Therefore, the difference in the type of study seems to be the main reason for the difference in results.

### Limitations

This study was conducted in a single educational center, and caution should be exercised when generalizing its findings. Additionally, due to the self-report nature of the questionnaires, the results may be subject to response bias. Furthermore, the participants'emotional state at the time of completing the questionnaires may have influenced the study's outcomes.

## Conclusion

The findings of the present study showed that the self-efficacy of the students'clinical performance is in a favorable state and their motivation is at a good level. However, the findings showed that the clinical education situation is at an average level. The findings of this study also indicated that motivation for choosing a field of study plays a significant role in predicting self-efficacy of clinical performance. Revision and development of executive policies in clinical education can be effective in improving the quality of clinical education. Because the quality of appropriate clinical education can guarantee the improvement of the self-efficacy of clinical performance and the training of competent human resources. Adopting motivational enhancement approaches and improving the quality of clinical education can contribute to the promotion of self-efficacy in the clinical performance of nursing students. It is suggested to evaluate the condition of students after graduation in cohort studies.

## Data Availability

Data supporting this study are available to you upon reasonable request from the corresponding author.
